# Kyste hydatique de la fosse cérébrale postérieure

**DOI:** 10.11604/pamj.2017.26.133.8363

**Published:** 2017-03-09

**Authors:** Abderrazzak El Saqui, Mohamed Aggouri, Mohamed Benzagmout, Khaled Chakour, Mohamed El Faizchaoui

**Affiliations:** 1 Service de Neurochirurgie, CHU Hassan II, Fès, Maroc

**Keywords:** Kyste hydatique cérébral, Fosse postérieure, chirurgie, Cerebral hydatid cyst, Posterior fossa, surgery

## Abstract

L'hydatidose est une parasitose cosmopolite endémique au Maroc. L'échinococcose cérébrale est rare représentant 1 à 2% de l'ensemble des hydatidoses de l'homme et la localisation auniveau de la fosse postérieure est exceptionnelle. Nous rapportons le cas d'un enfant âgé de 12 ansadmis pour syndrome d'hypertension intracrânienne évoluant depuis trois mois. La tomodensitométrie cérébrale a objectivé une formation kystique de la fosse postérieurene se rehaussant pas après injection du produit de contraste. L'intervention chirurgicale révélait un kyste hydatique. Ce diagnostic aété par la suite confirméparl'examen anatomo-pathologique. Le patient a reçuuntraitement médical à base d'albendazole en postopératoire. L'évolutionétait favorable six mois plus tard.

## Abstract (en)

Hydatidosis is an endemic affection in Morocco. Cerebral echinococcosis is a relatively rare entity accounting for only 1-2% of all hydatid cysts in humans. hydatid cyst of the posterior fossa is a very uncommon site for the disease. We report the case of a 12 year-old child admitted for high intracranial pressure. Brain CT scan showed a posterior fossa cyst without enhancement after contrast medium injection. Operative finding revealed a hydatid cyst. The histological examination of the tissue sample confirmed the diagnosis. The patient was given albendazole post operatively. He feels well six months later.

## Introduction

La pathologiehydatiqueesttrèsfréquente au Maroc et au pourtourméditerranéen [1]. La localisationcérébraleest rare, ne représenteque 2% environ de toutes les localisationshydatiques de l'organisme et touchesurtoutl'enfant et l'adolescent [2], la localization au niveau de la fosse cérébrale posterieur est exceptionnel. Les signes cliniques sont ceux d'un processus expansif intracranien, la TDM souventsuffisante pour poser le diagnostic, et le traitement reste chirurgical.

## Patient et observation

**Figure 1:**
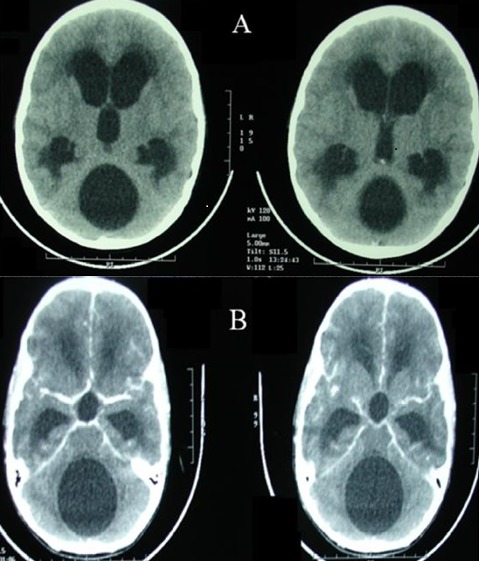
TDM cérébrale sans (A) et avec (B) contraste, qui objective un processuskystique de la fosse cérébralepostérieure, arrondi et homogène, ayant des contours bienlimités, sans œdèmepérilésionnelni de prise de contraste, associé à unehydrocéphalie active triventriculaire

Enfant de 12 ans, sans antécédents pathologiques notables, qui a présenté 3 mois avant son admission un syndrome d'hypertension intracrânienne fait de céphalées et de troubles visuels, compliqué ultérieurement de troubles de la marche. L'examen initial a retrouvé un patient conscient, apyrétique, ayant un syndrome cérébelleux stato-kinétique. L'examen ophtalmologique a objectivé un œdème papillaire stade III, l'acuité visuelle était réduite à 7/10 à droite et 8/10 à gauche. Une TDM cérébrale avec et sans contraste a été réalisée (Figure 1). Devant l'aspect scannographique typique, le diagnostic retenu était celui d'un kyste hydatique (KH) de la fosse cérébrale postérieure. En effet, l'interrogatoire a retrouvé que l'enfant qui est d'origine rurale était en contact étroit avec les chiens, et qu'il avait un frère de 21 ans opéré il y a 4 ans pour un kyste hydatique hépatique. Cependant, le bilan complémentaire, notamment l'échographie abdominale et la radiographie thoracique, n'a pas montré d'autres localisations associées. De même, la sérologie hydatique était négative. Le patient a été opéré par une voie sous-occipitale médiane. La corticotomie cérébelleuse prudente a permis la découverte d'une lésion kystique à paroi blanchâtre translucide rappelant l'aspect macroscopique d'un kyste hydatique encéphalique. L'extraction en bloc par hydrodissection s'est avérée dangereuse vu la survenue de deux épisodes de bradycardie lors de la tentative d'accouchement du kyste. Ceci nous a amené à réaliser une ponction aspiration première du kyste couplée à un lavage au sérum hypertonique, pour réduire le volume de la lésion avant l'ablation totale de la membrane kystique. Les suites opératoires immédiates étaient simples. La patiente a reçu un traitementmédical à base d'albendazole en postopératoire. Après un recul de 16 mois, l'examen neurologique est sans anomalies et le fond d'œil de contrôle a montré une régression totale de l'œdème papillaire au deuxième mois postopératoire.

## Discussion

L'hydatidose est une parasitose cosmopolite qui sévit encore à l'état endémique dans notre pays, constituant un réel problème de la santé publique. Habituellement observée au niveau du foie (60%) et des poumons (30%), la localisation encéphalique reste rare, ne dépassant pas 2% de l'ensemble des localisations somatiques [1]. L'enfant en est l'apanage (50-75%) vu la fréquence du contact avec les chiens, ainsi que les conditions d'hygiène précaire à cet âge notamment en milieu rural. L'âge moyen est de 5 à 8 ans [2]. Sur le plan physiopathologique, après son ingestion, et sous l'effet des sucs digestifs, l'embryon hexacanthe est libéré de sa coque protectrice, puis entreprend sa migration en s'introduisant dans les voies circulaires porte et lymphatiques des villosités. Sa plasticité l'autorise à franchir tous les capillaires. Le KH encéphalique siège préférentiellement à l'étage sus tentoriel, surtout au niveau du territoire de l'artère sylvienne. La localisation au niveau de la fosse cérébrale postérieure est exceptionnelle [3], le siège est variable: vermien [2], hémisphérique [4], pontique, intraventriculaire, et même extradural [5]. Morphologiquement, le KH encéphalique est sphérique et le plus souvent constitué d'une seule vésicule entourée d'un adventice mince, ce qui permet une excellente énucléation lors de l'intervention. Autour du kyste, la substance cérébrale est refoulée et atélectasiée avec peu de réaction gliale et pratiquement pas de réaction vasculaire.

La symptomatologie clinique, peu spécifique, associe de façon variable, un syndrome cérébelleux, des signes déficitaires, une paralysie des paires crâniennes. Contrairement à la localisation sus tentorielle, le syndrome d'hypertension intracrânienne est tardif, secondaire à la compression du 4^ème^ ventricule. Le scanner cérébral constitue l'examen clef. L'image caractéristique est celle d'un processus hypodense, bien limité, arrondi ou ovalaire, sans prise de contraste. Le diagnostic différentiel se pose avec l'astrocytomepilocytique et l'hémangioblastome. Cependant, dans ces lésions on note souvent la présence d'un bourgeon charnu prenant le contraste. Le kyste épidermoïde présente aussi un aspect hypodense mais des contours irréguliers. Le kyste arachnoïdien peut être également discuté. Toutefois, ce dernier est souvent de siège périphérique et sa forme n'est pas sphérique. Enfin, l'abcès présente une prise de contraste périphérique et une paroi épaisse qui le font distinguer du kyste hydatique [3]. L'imagerie par résonance magnétique est peu indiquée, et garde un intérêt dans les formes multiples pour faire l'inventaire de toutes les localisations en particulier celles qui peuvent passer inaperçues au scanner, ou encore, devant une localisation ou un aspect radiologique inhabituel: KH du tronc cérébral, KH calcifié, KH surinfecté, KH simulant une tumeur. e seul traitement radical des KHC demeure la chirurgie. La technique d'énucléation hydraulique d'AranaIniguez permet de séparer le KH du parenchyme cérébral et de l'accoucher progressivement par instillation du sérum salé à travers une sonde souple glissée entre le parenchyme et le kyste [6]. Cette méthode est simple et ne pose de problème que si le kyste est remanié ou siégeant au niveau des régions profondes, notamment le tronc, ou étroites comme la fosse cérébrale postérieure, car le volume surajouté lors de l'hydrodissection peut aggraver en per-opératoire la compression du tronc cérébral. Dans ces cas, et malgré le risque majeur de rupture et d'ensemencement cérébral, le recours à la méthode de ponction-aspiration de Digaammaimaginario [6] s'impose; elle consiste à ponctionner et à aspirer le contenu du kyste avant de procéder à l'ablation en masse de la membrane résiduelle.

## Conclusion

Le diagnostic du KH doit être gardé à l'esprit devant toute lésion purement kystique de la fosse cérébrale postérieure, notamment chez un enfant d'origine rurale. La TDM cérébrale est l'examen clef et le traitement est par excellence chirurgical. Cependant, l'éducation sanitaire, la lutte contre l'agent de transmission du parasite (les chiens errants) et l'inspection des viandes reste le seul moyen pour éradiquer cette pathologie.
